# Treatment for a primary multidrug-resistant B-cell acute lymphoblastic leukemia patient carrying a SSBP2-CSF1R fusion gene: a case report

**DOI:** 10.3389/fonc.2023.1291570

**Published:** 2023-11-30

**Authors:** Huan Wang, Yujiao Wang, Liangchun Hao, Xuan Liu, Jihong Zhang, Pin Yao, Danping Liu, Runan Wang

**Affiliations:** ^1^ Department of Pediatrics, Shengjing Hospital of China Medical University, Shenyang, Liaoning, China; ^2^ Precision Targeted Therapy Discovery Center, Institute of Technology Innovation, Hefei Institutes of Physical Science, Chinese Academy of Sciences, Hefei, Anhui, China

**Keywords:** Philadelphia chromosome-like acute lymphoblastic leukemia, children, multidrug resistance, SSBP2-CSF1R fusion, MRD

## Abstract

SSBP2-CSF1R is an important biomarker for clinical diagnosis and prognosis of Philadelphia chromosome-like acute lymphoblastic leukemia (Ph-like ALL). This case report presents a pediatric Ph-like ALL patient carrying the SSBP2-CSF1R fusion gene. The patient was resistant to most conventional chemotherapy regimens and to dasatinib, an inhibitor that has been reported to have a therapeutic effect on SSBP2-CSF1R fusion Ph-like ALL, as she remained minimal residual disease (MRD) positive (detection by flow cytometry) and SSBP2-CSF1R fusion gene (detection by RT-PCR) positive after five rounds of such regimens. We thus conducted a large-scale *in vitro* screening to assess the sensitivity of the patient’s leukemic cells to anti-cancer drugs. Based on the susceptibility results, we chose to combine cytarabine, homoharringtonine, dexamethasone, fludarabine, vindesine, and epirubicin for treatment. Clinical results showed that after a course of treatment, both MRD and SSBP2-CSF1R fusion gene turned negative, and there was no recurrence during an 18-month follow-up. In conclusion, our study suggests that the SSBP2-CSF1R fusion gene may be an important biomarker of primary drug resistance in Ph-like ALL, and indicate that the combination of cytarabine, homoharringtonine, dexamethasone, fludarabine, vindesine, and epirubicin can achieve optimal therapeutic results in this category of patients.

## Introduction

Acute lymphoblastic leukemia (ALL) is one of the most common malignancies in children. Although diagnosis and treatment through disease risk stratification has significantly improved the 5-year disease-free survival (DFS) and overall survival (OS) rates of ALL (1–3), 20% of patients are still prone to relapse (4). With the progress of genomics research, many prognostic genetic abnormalities involving BCR-ABL1, TEL-AML1, MLL translocation-related fusion genes, and colony-stimulating factor 1 receptor (CSF1R), among others, have been found in ALL (5–7).

CSF1R, also known as CD115 and M-CSF-R, belongs to the type III protein tyrosine kinase receptor family expressed on monocytes/macrophages, abdominal exudative cells, plasma cell-like and conventional dendritic cells, and osteoclasts (8–10). Natural ligands for this gene product include CSF1 and IL-34. Ligand binding to CSF1R causes its dimerization and activates downstream signaling pathways that play an important role in the differentiation and survival of monocytes and macrophages (11, 12). Various mutations and rearrangements involving the CSF1R gene have been described in a variety of diseases (13). Known translocation partner genes involved in CSF1R rearrangements identified in Philadelphia chromosome-like (Ph-like) ALL cases include SSBP2. Indeed, the SSBP2-CSF1R fusion, resulting from the translocation t(5;5)(q14;q32), is the most intensively characterized CSF1R fusion gene. However, clinical outcomes of ALL patient carrying the SSBP2-CSF1R fusion gene are variable (14, 15).

Herein, we present a case report of a primary multidrug resistant B-cell ALL patient carrying a SSBP2-CSF1R fusion gene. We summarize the clinical features of this patient and the efficacy of our treatment regimens, to provide reference for the management of pediatric B-ALL patients carrying this mutation.

## Case presentation

**Figure 1:**
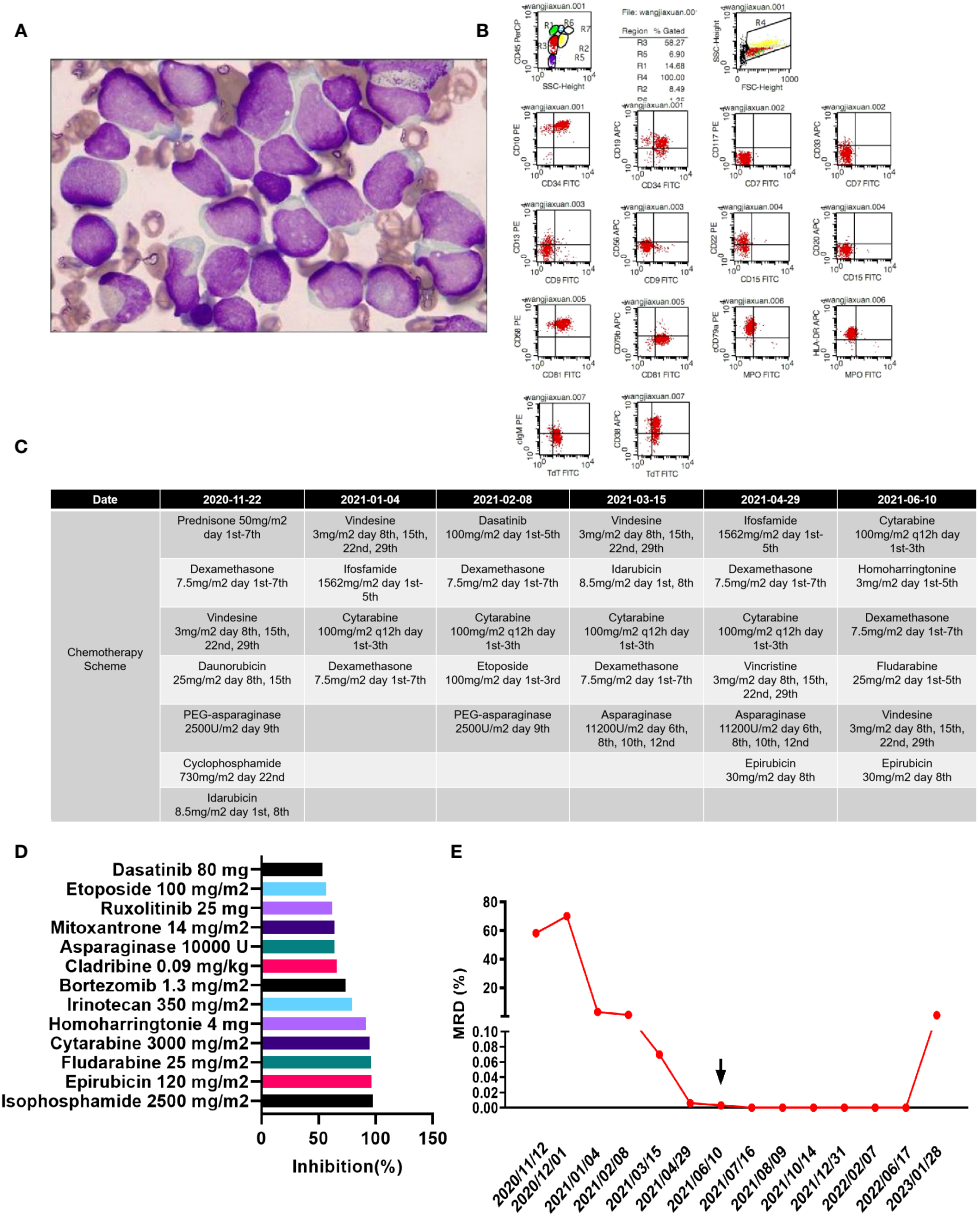
**(A)** Morphology of the patient’s lymphoblasts (scale: 10×). **(B)** Results of flow cytometry analysis. **(C)** Treatment flow chart. **(D)** Drugs with a relative inhibition rate higher than 50%. **(E)** MRD values during the treatment period.

A 4-year-old girl was admitted to our hospital on 12 November 2020 due to a 1-month history of paleness, fatigue, and fever. She had no family history of genetic disorders. Complete blood count (CBC) demonstrated a white blood cell count (WBC) of 31.35×10^9^/L, a hemoglobin (HGB) level of 4.6 g/L, and a platelet count of 21×10^9^/L. She was found to have a large spleen, extending to 4 cm below the costal margin, and an hepatomegaly. Bone marrow aspiration revealed lymphocyte hyperplasia. Predominance of prolymphocytes (78.2%) among total lymphoid cells indicated L2-type ALL in this patient (**Figure 1A**). Immunophenotyping *via* flow cytometry indicated that the blasts were mainly CD45min, positive for CD34, CD10, CD19, CD58, CD81, cCD79a, cTdT, and HLA-DR; partially positive for CD13, CD22, CD38, and cIgM; and negative for CD7, CD117, CD33, CD9, CD56, CD20, CD79b, and MPO, indicating a diagnosis of acute B lymphoblastic leukemia (B-ALL) (**Figure 1B**). No mutations in 236 commonly mutated genes in ALL were detected after performing next-generation sequencing (**Supplementary Table S1**). In turn, bone marrow Realtime-PCR (RT-PCR) analysis of 72 commonly detected fusion genes in leukemia identified the SSBP2-CSF1R gene fusion (**Supplementary Table S2**).

Chemotherapy schemes were conducted as shown in **Figure 1C**. The patient was induced with cyclophosphamide, vindesine, dexamethasone, idarubicin, PEG-asparaginase, daunorubicin (COILD), and triple intrathecal chemotherapy. She developed fever and facial swelling after induction therapy. Parotid routine computed tomography (CT) scan demonstrated swelling of the right parotid gland, and swelling and exudation of surrounding soft tissues. A chest CT demonstrated scattered pneumonia and consolidation of the lung tissue.

Dasatinib has been reported to benefit patients with the SSBP2-CSF1R fusion gene (16). However, because this patient developed a serious infection upon first treatment, we did not add dasatinib to the treatment regimen. On the 42th day after the first induction, there were still 3.08% blasts detected by flow cytometry in the bone marrow, suggesting resistance to routine chemotherapy. Thus, we adapted the induction regimen to include vindesine, ifosfamide, cytarabine, and dexamethasone. However, on the 34th day after the second induction, the patient still failed to achieve complete remission, with 0.96% blasts detected by flow cytometry remaining in the bone marrow. Since the infection was effectively controlled, we added dasatinib during subsequent treatment. The patient received two additional rounds of induction therapy but was still minimal residual disease (MRD) and SSBP2-CSF1R positive, as detailed in **Figure 1E**.

Consequently, we isolated leukemic cells from bone marrow aspirates and cultured them in ALL complete medium (Precedo, Hefei, China) for drug sensitivity analysis. Information on the drug panel and test results are listed in **Supplementary Table S3**. Drugs with an inhibition rate >50% are shown in **Figure 1D**. Finally, the patient received a regimen including cytarabine 156 mg/m^2^, days 1–5; homoharringtonine 3 mg/m^2^, days 1– 5, dexamethasone 7.8 mg/m^2^, days 1–7; fludarabine 25 mg/m^2^, days 1–5; vindesine 3 mg/m^2^, days 1 and 8; and epirubicin 30 mg/m^2^, day 8; and ultimately achieved complete remission both at the cellular level (MRD negative) and at the gene level (SSBP2-CSF1R negative). Then, two cycles of the same scheme were given as consolidation chemotherapies.

Due to family financial constraints, the patient did not receive a bone marrow transplant, and the parents chose the child to be discharged from the hospital for maintenance treatment (vindesine 3 mg/m^2^ day 1 + dexamethasone 8 mg/m^2^ dayss 1–5 + methotrexate 25 mg/m^2^ days 8, 15, and 22 + mercaptopurine 50 mg/m^2^ days 8–28). Upon regular follow-up (every 1–2 months), the patient remained MRD negative at follow-up on 29 August 2022 and was found to have relapsed at the follow-up examination on 28 January 2023. However, due to personal reasons, the patient was not treated and was lost to follow-up. The patient remained leukemia-free and MRD negative for 18 months from the last chemotherapy to the last follow-up.

## Discussion

ALL is a hematological malignancy driven by a genetic mutation that originates from hematopoietic stem/progenitor cells. Most patients with ALL present various cytological and molecular genetic abnormalities, which are important for guiding diagnosis, classification, treatment, and prognosis (17–19). With the development of more sophisticated screening technologies, in recent years, a new high-risk subtype of B-ALL, termed Philadelphia chromosome-like ALL (Ph-like ALL), has been discovered. Ph-like ALL is listed as a tentative type in the World Health Organization (WHO) 2016 classification of blood and lymphoid tissue tumors. Its gene expression profile is similar to Ph+ ALL, with a series of molecular abnormalities related to the activation of cytokine receptors and kinase signaling pathways (20–22). The Children’s Oncology Group trials, which included results from 1,725 patients with B-ALL, showed that the overall incidence of Ph-like ALL was 15.3% (264/1,725). Among these cases, the 5-year overall survival (OS) rates in high-risk children (aged 10–15 years, WBC > 50×10^9^/L), adolescents (aged 16–20 years), and young adult patients (21–39 years old) were 72.8%, 65.8%, and 25.8%, respectively (23). Another study of 798 adult patients with B-ALL showed that approximately 24.3% (194/798) of them had Ph-like ALL, with a 5-year OS rate of 23.8% compared to 52.4% for non-Ph-like ALL patients (24).

Fusion genes are important drivers of tumorigenesis and progression, often acting as atypically activated transcription regulators that promote the expression of proto-oncogenes, the inactivation of tumor suppressor genes, and/or the formation of new fusion proteins. These events trigger the activation or overactivation of downstream signaling pathways that promote cell growth, inhibit cell differentiation, and ultimately lead to tumorigenesis (25–27). At present, a variety of fusion genes, including SSBP2-CSF1R, have been found to be involved in the occurrence and development of ALL (28, 29). A study by Schwab et al. concluded that SSBP2-CSF1R fusions in childhood B-ALL are extremely rare, are restricted to Ph-like B-ALL patients, and are associated with variable outcome. Overall, their findings indicated that younger children tend to achieve longer DFS and experience later relapse after treatment, while older children may relapse more easily and earlier (15). It is possible that children with ALL who are SSBP2-CSF1R positive may benefit from the incorporation of tyrosine kinase inhibitors (TKIs) into their treatment regime in the early stages of their disease. Based on the case herein reported, which was not sensitive to commonly used regimens, we suggest that the SSBP2-CSF1R fusion gene may be an important cause of primary drug resistance in ALL. The results of *in vitro* tests showed that the patient-derived B-ALL cell line with positive SSBP2-CSF1R fusion gene was sensitive to imatinib and dasatinib, which further suggest that Ph-like B-ALL with CSF1R gene abnormalities may be treated with TKIs (30, 31). We thus used dasatinib combined with clinical treatment regimens, but did not observe very good effects, as exam results showed both continued MRD positivity and SSBP2-CSF1R expression.

With the development of precision medicine, personalized drug sensitivity screening for tumors has received increased attention from researchers (32). Cell-based drug susceptibility testing is a standard evaluation method for individualized tumor treatment. It is generally accepted that if the cytotoxic drug tested cannot kill tumor cells in the *in vitro* test system, it will not exert effective tumor suppression in the patient (33, 34). Based on this concept, we isolated leukemia cells from this patient, expanded them *in vitro*, and conducted large-scale drug susceptibility tests, hoping to find candidate drugs that effectively inhibited their proliferation. As a result, we chose to combine cytarabine, homoharringtonine, dexamethasone, fludarabine, vindesine, and epirubicin for treatment. Clinical results showed that after a course of treatment, both MRD and the SSBP2-CSF1R fusion gene turned negative. Additionally, no recurrence was observed during the subsequent 18-month follow-up.

In conclusion, our study suggests that the SSBP2-CSF1R fusion gene may be an important direct marker of primary drug resistance in Ph-like ALL. Combining cytarabine, homoharringtonine, dexamethasone, fludarabine, vindesine, and epirubicin is likely to achieve very good therapeutic outcomes in this category of patients. This study is expected to provide new insights into the treatment of Ph-like ALL patients with the SSBP2-CSF1R fusion gene.

## Data availability statement

The original contributions presented in the study are included in the article/**Supplementary Material**. Further inquiries can be directed to the corresponding author.

## Ethics statement

Written informed consent was obtained from the individual(s), and minor(s)’ legal guardian/next of kin, for the publication of any potentially identifiable images or data included in this article.

## Author contributions

HW: Investigation, Writing – original draft. YW: Resources, Writing – original draft. LH: Project administration, Writing – original draft. XL: Data curation, Writing – original draft. JZ: Data curation, Writing – original draft. PY: Formal Analysis, Writing – original draft. DL: Data curation, Writing – original draft. RW: Funding acquisition, Project administration, Supervision, Writing – original draft, Writing – review & editing.
