# Supplementary material for: Treatment for a primary multidrug-resistant B-cell acute lymphoblastic leukemia patient carrying a SSBP2-CSF1R fusion gene: a case report
Source: Front Oncol. 2023 Nov 30;13:1291570. doi: 10.3389/fonc.2023.1291570 (PMC10723836; doi:10.3389/fonc.2023.1291570)
Supplement: Supplementary file 1 [file Table_1.docx]

Supplementary table 1: 236 commonly mutated genes in leukemia were detected with Next generation sequencing assay

| *ABL1* | *ANKRD26* | *ARID1B* | *ARID2* | *ASXL1* | *ASXL2* |
| --- | --- | --- | --- | --- | --- |
| *ATM* | *B2M* | *BCL2* | *BCL6* | *BCOR* | *BCORL1* |
| *BIRC3* | *BLM* | *BRAF* | *BTK* | *CALR* | *CARD11* |
| *CBL* | *CCND1* | *CD28* | *CD58* | *CD79A* | *CD79B* |
| *CDKN1B* | *CDKN2A* | *CEBPA* | *CHD8* | *CREBBP* | *CSF3R* |
| *CXCR4* | *DDX41* | *DHX15* | *DKC1* | *DNMT3A* | *ELANE* |
| *EP300* | *EPOR* | *ETNK1* | *ETV6* | *EZH2* | *FBXW7* |
| *FLT3* | *FOXO1* | *GATA1* | *GATA2* | *GATA3* | *GFI1* |
| *GNA13* | *HAX1* | *ID3* | *IDH1* | *IDH2* | *IKZF1* |
| *IL7R* | *JAK1* | *JAK2* | *JAK3* | *KDM6A* | *KIT* |
| *KMT2A* | *KMT2C* | *KMT2D* | *KRAS* | *MAP2K1* | *MAPK1* |
| *MEF2B* | *MLH1* | *MPL* | *MSH6* | *MYD88* | *NF1* |
| *NOTCH1* | *NOTCH2* | *NPM1* | *NRAS* | *NTRK3* | *PAX5* |
| *PDGFRB* | *PHF6* | *PIGA* | *PLCG2* | *POT1* | *PPM1D* |
| *PRPF8* | *PTEN* | *PTPN11* | *RAD21* | *RB1* | *RHOA* |
| *RUNX1* | *SBDS* | *SETBP1* | *SF3B1* | *SH2B3* | *SMC1A* |
| *SMC3* | *SRP72* | *SRSF2* | *STAG2* | *STAT3* | *STAT5B* |
| *TCF3* | *TERC* | *TERT* | *TET2* | *TNFAIP3* | *TNFRSF14* |
| *TP53* | *TPMT* | *TRAF3* | *U2AF1* | *WT1* | *ZRSR2* |
| *ABCB1* | *ABCC3* | *ABCG2* | *ADSL* | *AKT2* | *AKT3* |
| *ALK* | *AMER1* | *ATRX* | *BCL11B* | *BMP7* | *CACNA1E* |
| *CACNA1G* | *CCDC168* | *CCND3* | *CDA* | *CECR2* | *CEP72* |
| *CHD2* | *CPA2* | *CRLF2* | *CSMD1* | *CTCF* | *CTLA4* |
| *CTNNB1* | *CUX1* | *CYBA* | *CYP2B6* | *CYP2C19* | *CYP2C8* |
| *CYP3A4* | *CYP3A5* | *DARS* | *DCTD* | *DHX30* | *DIS3* |
| *DNAH2* | *DNM2* | *DOK5* | *DROSHA* | *DYNC2H1* | *EGR2* |
| *ERCC1* | *ERG* | *EVI1* | *FAM46C* | *FAT1* | *FCGR3A* |
| *FGFR1* | *GNAS* | *GSTM1* | *GSTP1* | *HIST1H1E* | *HLA-DRB1* |
| *IL2RB* | *IMPDH2* | *IRF4* | *ITPA* | *KDM5C* | *KDM6B* |
| *LINC00251* | *MACF1* | *MAP3K7* | *MED12* | *MTHFR* | *MTRR* |
| *MYC* | *NF2* | *NFATC2* | *NFKBIA* | *NFKBIE* | *NR3C1* |
| *NSD2* | *NT5C2* | *NTRK1* | *NUDT15* | *PCLO* | *PDGFRA* |
| *PIK3CA* | *PIK3R1* | *PNPLA3* | *PRKDC* | *PROX1-AS1* | *PRPS1* |
| *RIT1* | *ROBO1* | *ROBO2* | *ROBO3* | *RPL10* | *RPS15* |
| *RRM1* | *RRM2* | *RRM2B* | *SAMHD1* | *SERPINE1* | *SETD2* |
| *SF1* | *SLC22A1* | *SLC29A1* | *SLCO1A2* | *SLCO1B1* | *SMAD4* |
| *SOCS1* | *SOD2* | *SOS1* | *SOX11* | *SPI1* | *SRCAP* |
| *STAG1* | *STAT5A* | *STAT6* | *STIM1* | *SUZ12* | *TNF* |
| *TRIM24* | *UGT1A1* | *UGT1A8* | *USH2A* | *USP7* | *XPO1* |
| *XRCC5* | *ZMYM3* |  |  |  |  |

Supplementary table 2: 72 common fusion genes in leukemia were detected with RT-PCR assay

| *BCR-ABL* | *TEL-PDGFRB* | *SIL-TAL1* | *FIP1L1-PDGFRA* | *E2A-HLF* | *AML1-MDS1* |
| --- | --- | --- | --- | --- | --- |
| *AML1-MTG16* | *TEL-AML1* | *CBFB-MYH11* | *MLL-AF4* | *DEK-CAN* | *E2A-PBX1* |
| *NPM-MLF1* | *AML1-ETO* | *TEL-ABL* | *MLL-AF9* | *ETV6-PDGFRA* | *PML-RARA* |
| *NUP98-HOXA13* | *NUP98-HOXC11* | *NUP98-HOXD13* | *NUP98-HOXA9* | *NUP98-HOXA11* | *NUP98-PMX1* |
| *MLL-AF6* | *MLL-AF10* | *MLL-ELL* | *MLL-ENL* | *MLL-AF17* | *MLL-AF1q* |
| *MLL-AF1P* | *MLL-AFX* | *MLL-SEPT6* | *PLZF-RARA* | *STATB-RARA* | *TEL-JAK2* |
| *NPM-RARA* | *FIP1L1-RARA* | *PRKAR1A-RARA* | *NUMA1-RARA* | *NPM-ALK* | *TLS-ERG* |
| *SET-CAN* | *ETV6-ABL1* | *RCSD1-ABL1* | *RSCD1-ABL2* | *MYB-TYK* | *NUP214-ABL1* |
| *MYH9-IL2RB* | *PAX5-JAK2* | *CRLF2* | *IKZF1* | *PAG1-ABL2* | *SSBP2-JAK2* |
| *ZMIZ1-ABL1* | *STRN3-JAK2* | *TERF2-JAK2* | *SSBP2-PDGFRB* | *TNIP1-PDGFRB* | *ZEB2-PDGFRB* |
| *BCR-JAK2* | *EBF1-JAK2* | *SSBP2-CSF1R* | *ETV6-JAK2* | *PPFIBP1-JAK2* | *SNX2-ABL1* |
| *ETV6-NTRK3* | *ZC3HAV1-ABL2* | *EBF1-PDGFRB* | *TPR-JAK2* | *RANBP2-ABL1* | *ATF7IP-JAK2* |

Supplementary table 3: The result of in vitro drug sensitivity screening

| **Drugs** | **Classification** | **Dosage** | **Concentration** | **Inhibition rate (%)** |
| --- | --- | --- | --- | --- |
| Bortezomib | molecular targeted therapeutic drugs | 1.3 mg/m^2^ | 0.083 μM | 73.65 |
| Irinotecan | chemotherapy regimens | 350 mg/m^2^ | 0.153 μM | 79.46 |
| Cytarabine | chemotherapy regimens | 100 mg/m^2^ | 0.2601 μM | 75.38 |
|  |  | 2000 mg/m^2^ | 5.202 μM | 92.9 |
|  |  | 3000 mg/m^2^ | 7.803 μM | 94.81 |
| Fludarabine | chemotherapy regimens | 25 mg/m^2^ | 2.68 μM | 95.91 |
| Etoposide | chemotherapy regimens | 60 mg/m^2^ | 0.6 μM | 43.3 |
|  |  | 100 mg/m^2^ | 1.002 μM | 56.46 |
| Cladribine | chemotherapy regimens | 0.09 mg/kg | 0.0018 μM | 65.88 |
| Asparaginase | other | 6000U | 4440 U/L | 63.42 |
|  |  | 10000U | 7400 U/L | 64 |
| Vincristine | chemotherapy regimens | 1.4 mg/m^2^ | 0.0158 μM | 47 |
| Isophosphamide | chemotherapy regimens | 1200 mg/m^2^ | 256.92 μM | 83.96 |
|  |  | 2500 mg/m^2^ | 535.25 μM | 97.62 |
| Dasatinib | molecular targeted therapeutic drugs | 80 mg | 0.011 μM | 53.32 |
| Decitabine | demethylation | 15 mg/m^2^ | 0.33 μM | 15.86 |
|  |  | 20 mg/m^2^ | 0.44 μM | 22.02 |
| Cyclophosphamide | chemotherapy regimens | 10 mg/Kg | 1.98 μM | 29.77 |
|  |  | 15 mg/Kg | 2.97 μM | 48.17 |
| Ruxolitinib | molecular targeted therapeutic drugs | 15 mg | 0.02 μM | 52.9 |
|  |  | 20 mg | 0.027 μM | 55.84 |
|  |  | 25 mg | 0.0348 μM | 62.12 |
| Nilotinib | molecular targeted therapeutic drugs | 230 mg | 0.021 μM | 15.48 |
| Methylprednisolone | glucocorticoid | 40 mg | 0.38 μM | 19.67 |
| Rituximab | antibody | 375 mg/m^2^ | 500 ug/ml | 6.94 |
| Topotecan | chemotherapy regimens | 1.25 mg/m^2^ | 0.064 μM | 40.7 |
| Epirubicin | chemotherapy regimens | 60 mg/m^2^ | 1.54 μM | 94.26 |
|  |  | 120 mg/m^2^ | 3.08 μM | 96.3 |
| Mitoxantrone | chemotherapy regimens | 8 mg/m^2^ | 0.15 μM | 54.77 |
|  |  | 14 mg/m^2^ | 0.27 μM | 63.87 |
| Arsenic trioxide | chemotherapy regimens | 7 mg/m^2^ | 4.795 μM | 34.53 |
| Homoharringtonie | chemotherapy regimens | 1 mg | 0.03 μM | 88.93 |
|  |  | 4 mg | 0.14 μM | 91.26 |
